# Integral Histogram with Random Projection for Pedestrian Detection

**DOI:** 10.1371/journal.pone.0142820

**Published:** 2015-11-16

**Authors:** Chang-Hua Liu, Jian-Kun Lin

**Affiliations:** Department of Medical Imaging, The 174th Hospital of Chnese PLA, The Succeed Hospital affiliated to Xiamen University, Xiamen, Fujian Province, China; Xiamen University, CHINA

## Abstract

In this paper, we give a systematic study to report several deep insights into the HOG, one of the most widely used features in the modern computer vision and image processing applications. We first show that, its magnitudes of gradient can be randomly projected with random matrix. To handle over-fitting, an integral histogram based on the differences of randomly selected blocks is proposed. The experiments show that both the random projection and integral histogram outperform the HOG feature obviously. Finally, the two ideas are combined into a new descriptor termed IHRP, which outperforms the HOG feature with less dimensions and higher speed.

## Introduction

The HOG [1] feature has became one of the most popular descriptors in object recognition since being proposed in 2005. The advantage of the HOG feature is that it’s not sensitive to small shift and illumination change, so it can encode the edge information of the object efficiently. This is due to its oriented-gradients based, normalized histogram extracted from (overlapped) blocks for description. However, several issues of HOG retain open, i.e.: (1) the feature dimension and the computing cost are high. (2) It performs poor in occlusions. (3) The other region-based information are lack, such as color, shape and texture.

In order to improve the computation efficiency, Zhu et al. [2] allowed the blocks to vary somewhat in size and used the Integral Histogram [3] to calculate the HOG feature quickly. The distinctive blocks are chosen by Adaboost to build a cascaded classifier. The speed of detection is about 70 times quicker than the original HOG feature with the comparable accuracy. Zhang et al. [4] and Pedersoli et al. [5] applied the HOG feature at multiple resolutions in which both the accuracy and efficiency are improved. Wojek et al. [6] used the parallel technique to implement the HOG on GPU, upon which a real-time pedestrian detection system is built.

To overcome occlusion, Dalal [7] used the part-based pedestrian detection by dividing the body into head-shoulder, torso and legs. The HOG features of the three parts are used to build the classifier together. In such a case, a missing part will not result in the detection failure, and therefore the occlusion can be tackled to a certain degree. Wang et al. [8] analyzed the response of each block to find the occluded blocks by decomposing the output of SVM classifier, and then local binary feature was further include to be a complement of HOG.

To enrich the description of HOG, Dalal [7] combined the HOG feature with the histogram feature based on movement and appearance for pedestrian detection in videos. Schwartz et al. [9] combined the HOG feature with color, texture and edges to create the considerable diversity descriptor. They also used the Partial Least Squares (PLS) to reduced the dimension of descriptor, which has shown the lower error rate than the original HOG descriptor. Watanabe et al. [10] proposed a co-occurrence Histograms of Oriented Gradients, to adapt the contextual statistics into the HOG feature.

Although many improvements of the HOG feature have been proposed, there is few work taking deep insights into the effects of orientation and magnitude of the gradient, which is the fundamental step in building the HOG descriptor. In this work, we will show that, instead of spacing the orientation bins evenly, the gradients can be projected by a random matrix and a regular polyhedron respectively, which surprisingly outperforms the original HOG descriptor with the similar dimension. Our work indicates that the gradient magnitude plays a crucial role in HOG. Besides, dividing the blocks into cells can improve the descriptor accuracy, as it describes more details.

In the HOG feature, the blocks are determined in the fixed size and positions; Based on the rigid global gradient, HOG is hard to capture the contour information of pedestrian precisely, since the training examples may not be aligned well. Inspired by the compressive sensing theory and other variants of HOG [11][12], especially its application on tracking [13], an improvement encoding scheme based on the integral histogram based improvement is proposed. Rather than using the global gradient, the new descriptor is based on the differences among local blocks. In each iteration, two blocks are chosen in the random positions and the difference between the two blocks is measured. In the end, differences all around iterations are combined to form the final descriptor. The experiments show that the integral histogram not only reduces the dimension significantly, but also outperforms the original HOG feature.

As discussed, the random projector and difference of local blocks can improve the performance respectively. Therefore, an Integral Histogram with Random Projection (IHRP) descriptor are proposed. In the novel descriptor, the gradients are projected by a random matrix and then the integral histogram is calculated as the feature. The experiments show that the proposed descritptpr outperforms the HOG with a low dimension and a fast speed. It can also perform well in the case of partial occlusions.

The rest of the paper is organized as follows: Related works on pedestrian detection are presented in Section 2. Section 3 describes the algorithms of random projection and the integral histogram. The experimental results are presented in Section 4. Finally, Section 5 concludes the paper.

## Related Works

Pedestrian detection is still an active research field in computer vision, new benchmarks [14][15] and novel detection methods [16][17][18] are proposed recently. There also exist some review papers [19][20][21] on pedestrian detection. Generally speaking, a full pedestrian detection system for intelligent vehicle includes the six following module [19]: pre-processing, candidates generation, classification, verification and refinement, tracking and application. Among these modules, classification is the core component, which involves vision and machine learning. Broadly speaking, classification consists of feature extraction and classifier. The former deals with descriptors, while the latter provides algorithms to learn from labeled samples based on the descriptors.

A pedestrian classifier decides if a candidate window contains a pedestrian or not. Popular supervised classifiers used in pedestrian detection are different types of neural networks (e.g. multi-layer perceptron, convolution neural network, deep learning, etc.), AdaBoost variants (e.g. Real AdaBoost, LogitBoost, GentleBoost, MPLBoost, etc.), support vector machine (SVM; linear, kernelized [22], latent [23], etc.), Random Forest [24], et al. In this section, we only briefly review the feature extraction of pedestrian detection.

Roughly speaking, feature for pedestrian detection can be divided into three categories: low-level feature, high-level feature, learning-based feature.

The commonly used low-level feature are color and texture. For example, Oren et al.[25] introduce the basic Haar features, which is extended by Viola and Jones [26] and called Haar-like feature set. Haar feature, AdaBoost, and integral image are three key intergradient for the first real-time face detection system. Levi et al. [27] introduce the Edge Orientation Histogram (EOH) features to compensate for Haar-likes ones, which only rely on gradient information. Dalal and Triggs [7] proposed another gradient-based features, called Histogram of Oriented Gradient, which is the most popular ones nowadays. Local Binary Pattern (LBP) proposed by Ojala et al. [28], is very successful in texture classification. Wang et al. [29] combined the uniform LBP and HOG features, where the LBP histograms are computed based on the cell grid used for HOG features. The computational cost of LBP and HOG is high, so Hinterstoisser et al. [30] proposed the dominant orientation template features, which can be used as an alternative to HOG with similar detection performance, but being about two orders of magnitude faster. People are with different kind of clothes, so color is not distinctive for pedestrian detection. But color self-similarity between different blocks in the scanning window is somewhat powerful.

Here the high-level feature denotes the high order statistical information of low-level feature. Watanabe et al. [31] proposed Co-occurrence HOG (CoHOG), which can be seen as second order of HOG feature. A major problem of CoHoG is of huge dimension, so Hiromoto et al. [32] divide the CoHOG feature into many smaller ones, and a rejection-cascade classification framework is constructed based on the smaller vectors. Tuzel et al. [33] proposed a fast region descriptors for object detection, which is called covariance features. Actually covariance matrices form a Riemannian manifold, not the commonly used vector space. So Tuzel et al. [33] use the LogitBoost classifier with weak classifiers operating in the tangent space of the manifold.

Learning-based feature, also called data-driven feature or feature mining, denotes the algorithms that learn the descriptors as part of the process of learning the pedestrian classifier, which is the realm of artificial neural networks. Enzweiler et al. [34] evaluated the pedestrian classification performance based on a feed-forward neural network with local receptive field (LRF) in the hidden layer. Sermanet et al. [35] proposed a method using unsupervised convolutional sparse auto-encoders to pre-train features, and end-to-end supervised learning to train the a pedestrian classifier.

## The Improvement of HOG

In the HOG [1] feature, the orientations are divided into 9 uniform bins, and the histograms of gradient are calculated by weighting the magnitude into corresponding bins according to the orientation. It can be seen as the uniform projecting. Besides, the blocks are overlapped and a global feature is generated finally.

### 3.1. Random Projection

In this paper, the random projectors are considered firstly. A random matrix *M*_*g×k*_ composed of random numbers is used for projecting the gradient magnitude. It means the gradient directions are randomly divided into uneven bins.

The random matrix is generalized as follows: Firstly an initial matrix *M*_*g×k*_ is generalized, where *g* represents the number of gradient components and *k* represents the projection dimension in a block, with *k* << *M*. Each element in the matrix is set to a random number (between -128 and 128), and then a normalization according to column is performed.

Oreifej et.al [11] used the gradient magnitude as an extra dimension and formed a proposed 4D normal. Therefore they can select different bins from different gradient magnitude and achieved the state of the art in recognition accuracy. Inspired by this, we define the 3D normal of the pixel as

$$N=\frac{1}{\sqrt{dx^{2}+dy^{2}+1}}\left(\begin{array}{l} dx\\ dy\\ -1 \end{array}\right),$$ (1)

where $\left(\begin{array}{l} dx\\ dy \end{array}\right)$ is the gradient of the pixel.

The 3D normal can also projected by the random matrix with *g* = 3. The process of random projector is showed as Algorithm 1.

**Algorithm 1: Random Projection Feature**

**Input:** Image I(x, y), the number of gradient components *g*, the projection dimension in a block *k*.

**Output:** Random Project Feature *F*

1. Generate a matrix *M*_*g×k*_. Each element of the matrix is formed by a random number (from -128 to 128). g∈{2,3}, *k* = 5, 10, 15…

2. Calculate each pixel’s gradient $\left(\begin{array}{l} dx\\ dy \end{array}\right)$, and get the normal vector

$$N(\text{x},\text{y})\;=\;\{\begin{array}{l} \frac{1}{\sqrt{dx^{2}\;+\;dy^{2}}}\left(\begin{array}{l} dx\\ dy \end{array}\right)\;if\;G\;=\;2\\ \frac{1}{\sqrt{dx^{2}\;+\;dy^{2}\;+\;1}}\left(\begin{array}{l} dx\\ dy\\ -1 \end{array}\right)\;if\;G\;=\;3 \end{array}$$

3. Multiply normal vector *N*_*1×G*_(x,y) and each column of the matrix *M*_*g×k*_, get each pixel’s projected value *P*_1×k_ = (p_1_,p_2_,…,p_*i*_,…,p_*k*_), if *p*_*i*_<0 then *p*_*i*_ = 0.

4. Refer to [1], vote all pixels’ projected value into uneven *bin*_i_(*i* = 1,2,…,*k*) for each block, and do normalization.

5. Conbined each block’ bins as the final random projection feature *F*.

Besides, the regular geometric objects called polyhedrons are considered to quantize the 3D space (seen as 3.1.3).

### 3.2. Integral Histogram

In traditional HOG, the location and the size of the block are fixed. The template needs to be regenerated in the case of occlusions, global shift and some large offsets of parts. In order to overcome this over-fitting problem, the random blocks of random size and position are selected through several iterations. In this way, the inflexible block sampling of template can be avoided, making features more robust to the above changes.

**Algorithm 2: Random Integral Haar Feature**

**Input:** Image I(x, y), a matrix *M*_*x×y*_, Maximum Iterations *n*, Number of bins *m*.

**Output:** Random Integral Haar Feature *F*.

1.Calculate gradient of each pixel, where G(x, y) as gradient magnitude, Θ(x, y) as gradient direction.

2.Build matrix *M*_*x×y*_ for each interval, vote each pixel’s gradient magnitude according to Θ(x, y).

3. Calculate each matrix *M*_*x×y*_’s integral graph, denoted by *M*_*(x+1)×(y+1)*_.

4. **for** *i* = 1,2,…*n*

**for** *j* = 1,2,…*m* **do**

  Randomly generated two rectangles *A* and *B*, and calculate the sum of gradients for each rectangle respectively, denoted as *sum*_*A*_,*sum*_*B*_,

**If** *sum*_*B*_<*sum*_*A*_

*sum*_*A*_ = *sum*_*B*_;

  put *sum*_*A*_ into *F*.

**end for**

**end for**

Normalize for each *M*_*(x+1)×(y+1)*_.

**Return** final random integral haar feature *F*.

}

In addition, the differences between local blocks are more powerful than the global block to describe the information in object recognition. It can better describe the difference between the histograms. There are many ways to describle the difference between two local blocks. Empircially, the performance using MIN*(A*, *B)* is superior to the others (*A* and *B* represents two different histogram respectively). Since the minimun of two histograms can better capture the main information of two blocks. The process of Integral Histogram is given as Algorithm2.

## The Experimental Results

In this section, the random projection and integral histogram are evaluated respectively, and the proposed IHRP descriptor is compared with the HOG. The evaluation dataset is the entire INRIA dataset [1]. The size of each sample is 64*128. 2,416 of the images were selected as the positive training examples while 12,180 as the negative ones. All algorithms are tested with 1,126 positive examples and 30,000 negative examples.

### 4.1. Random Projection

Firstly, the random matrix projectors with different dimension (*k*) are evaluated. The influence of cells is also considered. Inspired by [11] and [12], a 3D normal projection by polyhedron and random matrix are evaluated.

#### 4.1.1 The influence of the dimension of random matrix

**Fig 1:**
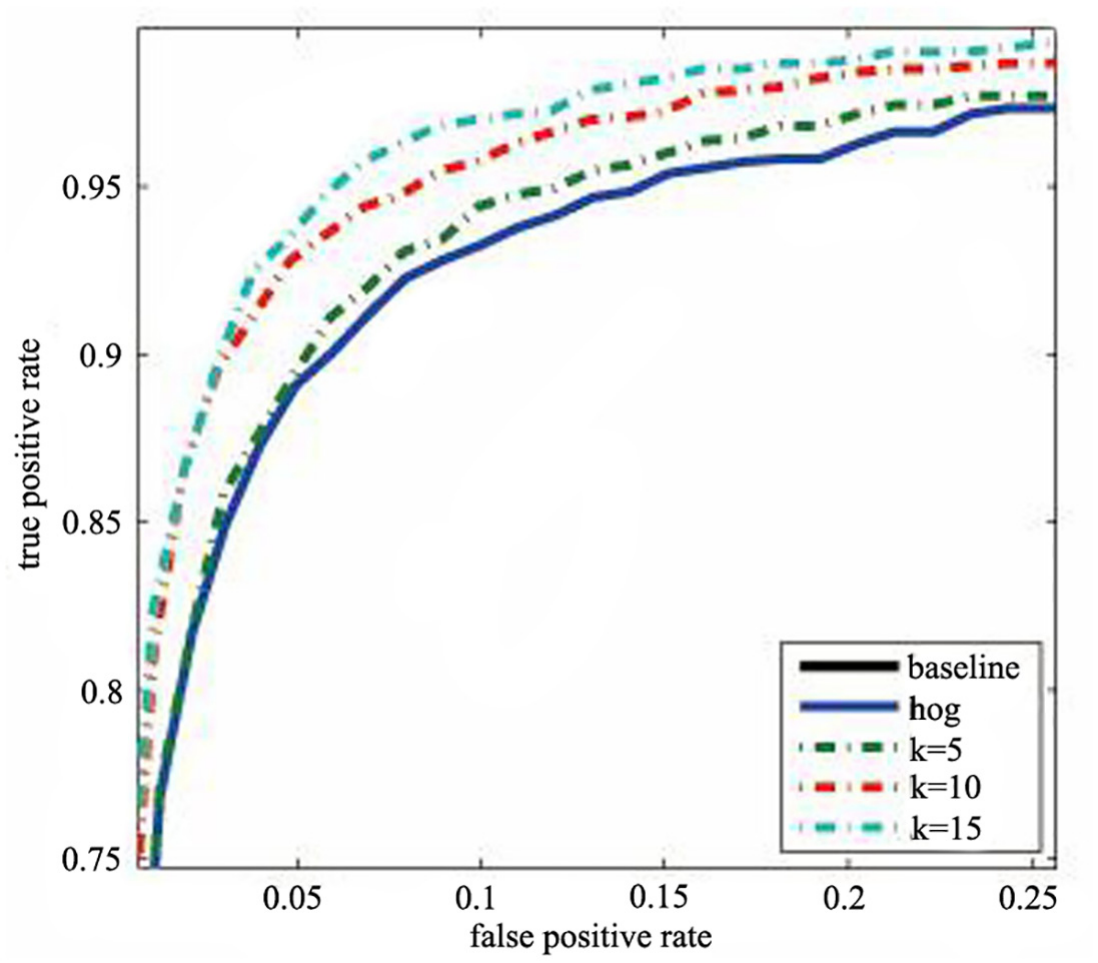
The performance of selected *k*.

The ROC curves of selected *k* on INRIA dataset [1] are showed as Fig 1, where *k* is dimension of random matrix. It shows the random projection outpefroms the HOG feature with less dimensions. As the value of *k* increases, the performance will be improved significantly, with a turning point at *k* = 10. Finnaly the number of random numbers is set to 10, which is similar to the 9 bins as the HOG suggested. But our algorithm is much better in accurancy.

#### 4.1.2 The influence of cells

**Fig 2:**
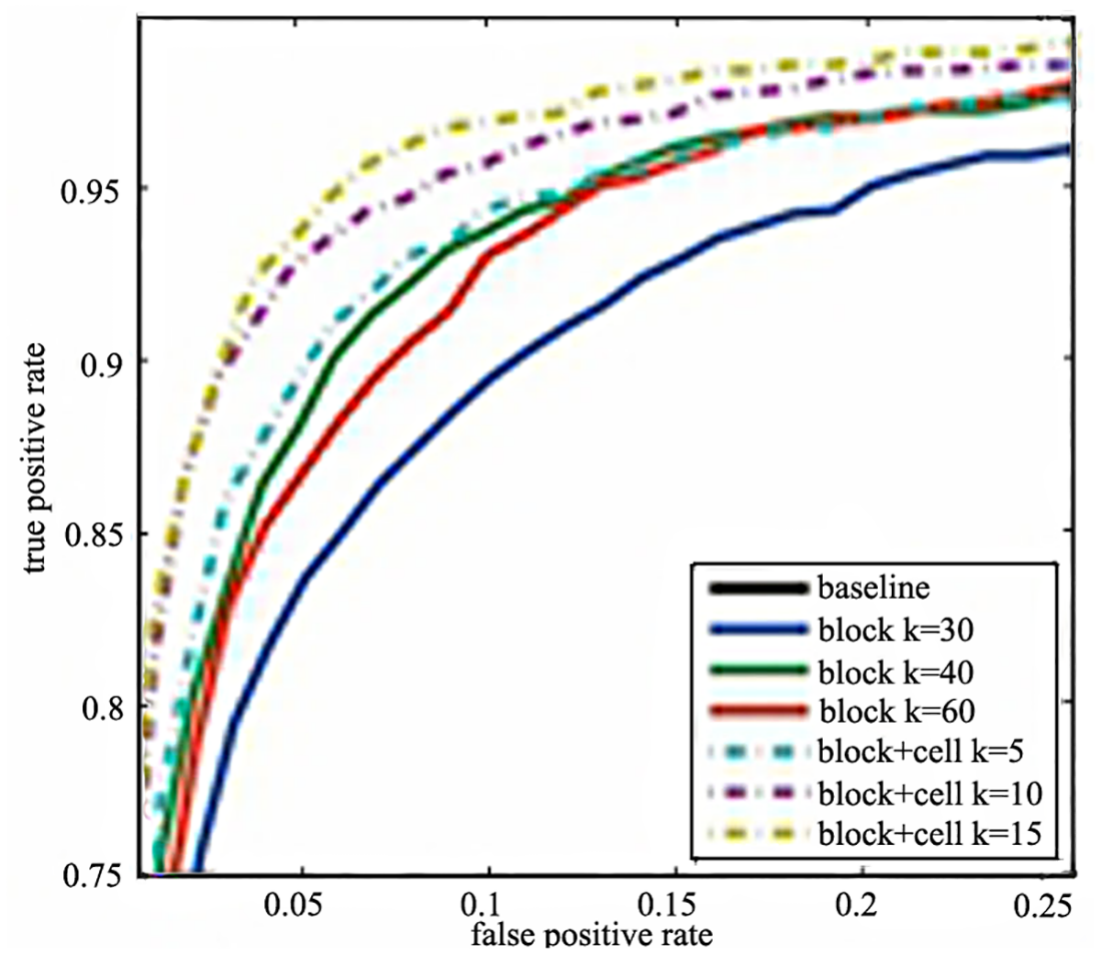
The performance of whether using cells on INRIA dataset.

The performances of whether the cells are used in the same dimension are showed as Fig 2. The ‘cell+block *k* = 5’ means that the gradient orientations are projected into 5 histograms, and the dimension is 4*5*105 = 2,100 which is the same with ‘block *k* = 20’ (20*105). It’s obvious that the cells can improve the accuracy dramatically.

#### 4.1.3 3D normals

**Fig 3:**
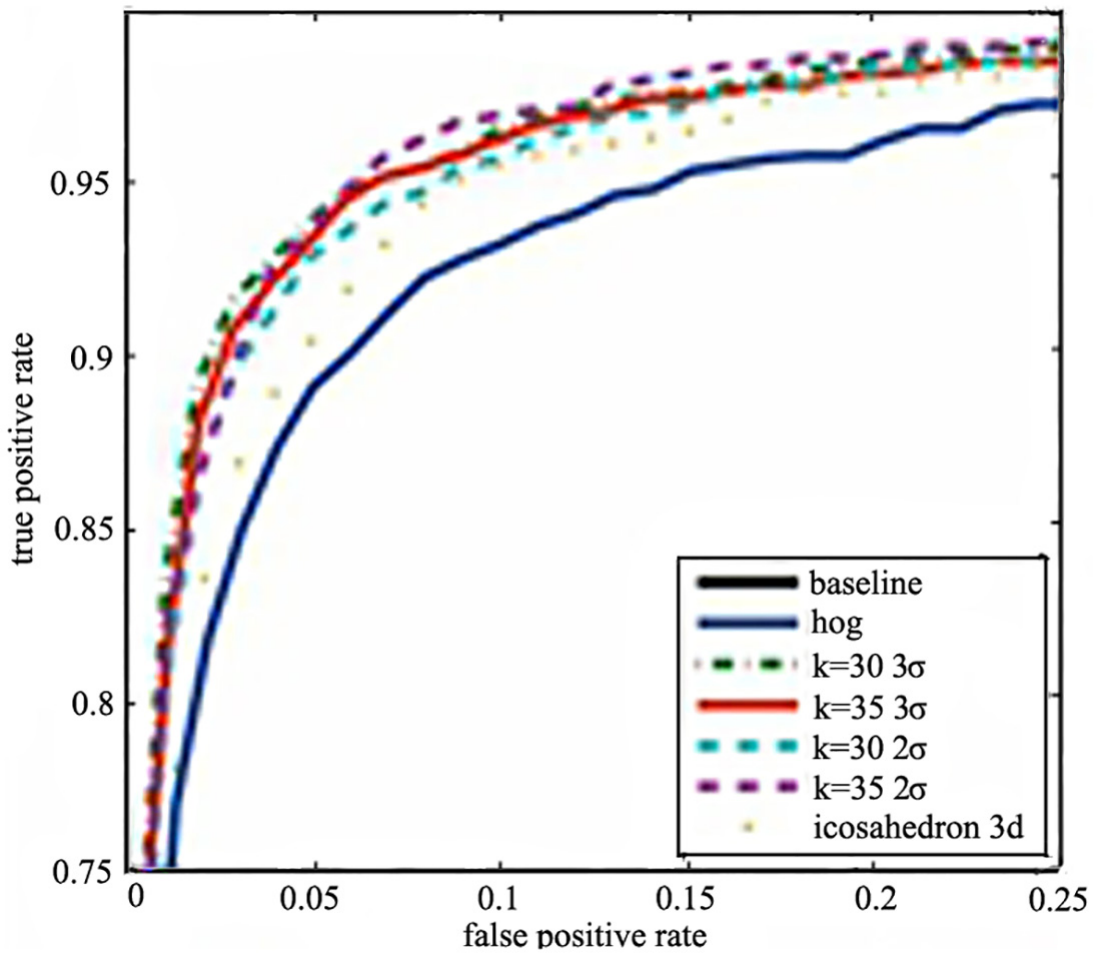
The performance of 3D normals projected by polyhedron and random matrix.

In this section, the 3D normals projected by polyhedron and random matrix are showed as Fig 3. In particular, the icosahedron with 12 vertices is used and the vertices are given as: (0, ±1, ±φ), (±1, ±φ, 0), and (±φ, 0, ±1), where 1/φ = 2 /(1 + √5) is a constant called the golden ratio. The quantization of 3D space by random projector is showed as Fig 3 (*k* = 10 3d, *k* = 15 3d). It shows that the 2D gradient combined with *k* = 15 perform best. The second one is the 3D normals with *k* = 10. The 3D normals projected by polyhedron (icosahedrons 3d) perform worse than the random projector, but still outperforms HOG feature. It shows that the random projectors with the proper dimension can achieved the better accuracy whether using 2D gradient or 3D normals.

### 4.2. Integral Histogram

**Fig 4:**
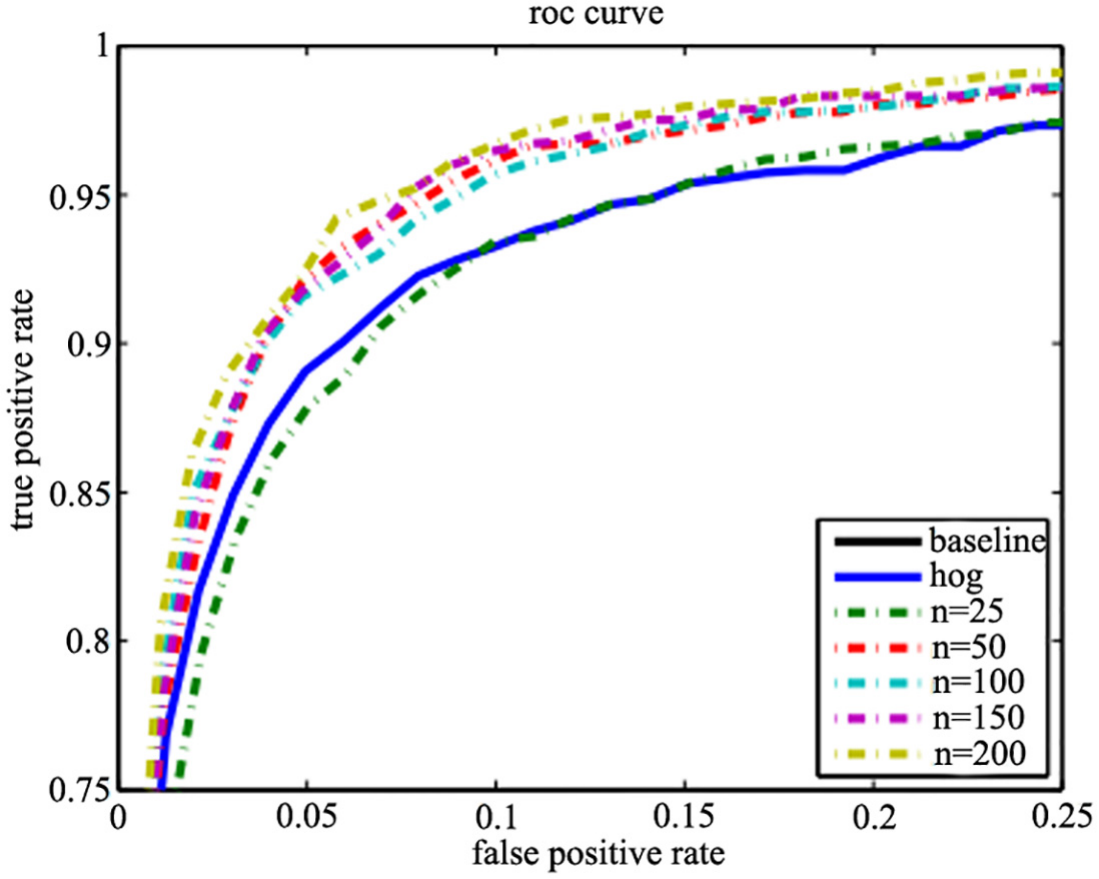
The performance of integral histogram with different number of block pairs *n*.

The performances of the selected number (*n*) of the random block pairs with cells are showed as Fig 4. In general, the performances will be better when *n* increase. The performance is similar to the HOG feature when *n* = 25. And it grows rapidly when *n* increases from 25 to 50 but not obviously when *n* is larger than 50. So *n* is seleted as 50 and 100 respectively.

### 4.3. Integral Histogram with Random projector

**Fig 5:**
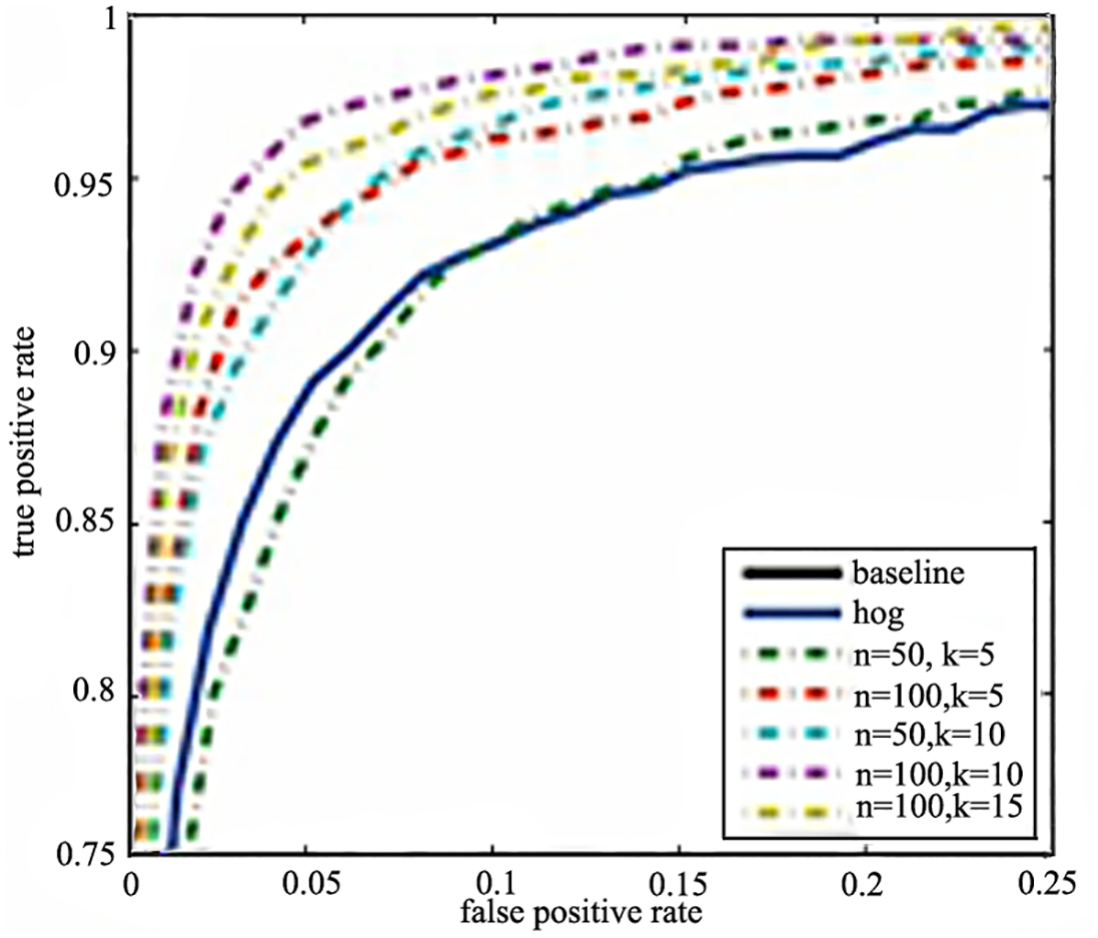
The performance of the proposed integral histogram with random projector (IHRP) with different *n* and *k*.

The combination of integral histogram with random projector is also considered. In particular, the cells are used to better describe the detail. The performances are showed in Fig 5. It shows that our proposed descriptor outperforms the HOG with a low dimension. It performs equally strong to the HOG descriptor with a quarter of HOG’s dimension when *n* = 50 and *k* = 5 (50*5*4 = 1000 dimensions). As the dimension increases to half of HOG when *n* = 50 and *k* = 10 (50*10*4 = 2000 dimensions), it outperforms much than the HOG at 2 times speed. Our method reaches the best performance when *n* = 100 and *k* = 10 (100*10*4 = 4000 dimensions). In the best case, the number of dimensions is similar to that of the HOG, but the accuracy and the efficiency is much better.

**Fig 6:**
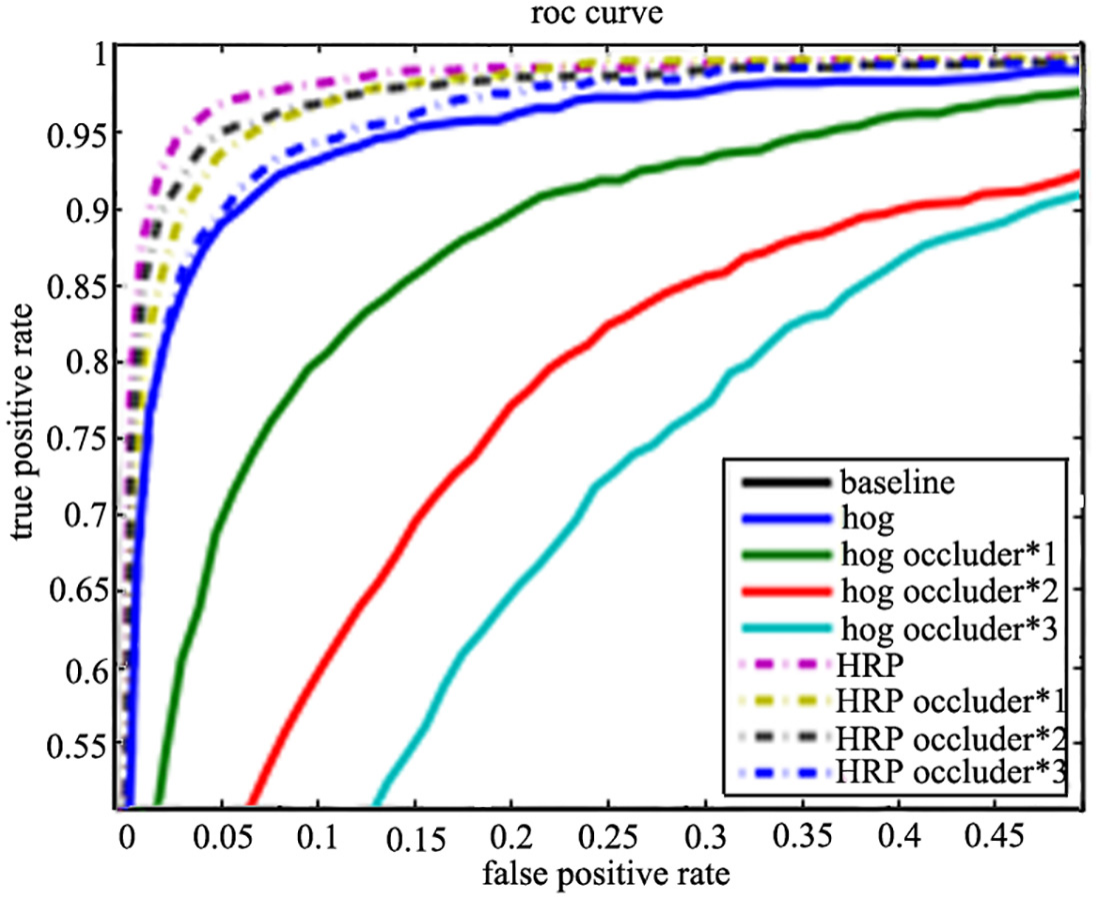
The performance of the proposed IHRP in the case of occlusions.

In order to evaluate the performance in the situation of occlusions, each example in the test set is embedded with the blocks of size 16*16 in random position. The number of blocks can be one, two or three. As it shows in Fig 6, the proposed method outperforms HOG much better when *n* = 100 and *k* = 10. Besides, as the number of local blocks pairs grows, the performances will be improved further.

## Conclusions

In this paper, the orientation and magnitude of gradient are studied and an integral histogram is proposed. The experiments show that both the random projector and integral histogram outperform the HOG feature obviously. Especially, the orientations can be randomly projected by random matrix and polyhedron. Besides, the local difference descriptor based on the integral histogram is more flexible and robust to the global HOG feature. Finally, the two ideas are combined to form the new feature IHRP, which can outperform the HOG feature with lower dimension and higher speed even in the case of partial occlusions.

In the future, a deep study of local blocks will be investigated, such like using AdaBoost to choose the more distinctive blocks for the templates.
